# Effects of Astragaloside IV on treatment of breast cancer cells execute possibly through regulation of Nrf2 via PI3K/AKT/mTOR signaling pathway

**DOI:** 10.1002/fsn3.1154

**Published:** 2019-09-18

**Authors:** Xiao‐Qing Zhang, Chang Yao, Wei‐He Bian, Xu Chen, Jing‐Xian Xue, Zhi‐Yuan Zhu, Yu Ying, Yan Lei Xu, Cong Wang

**Affiliations:** ^1^ The Affiliated Hospital of Nanjing University of Chinese Medicine Nanjing China

**Keywords:** breast cancer, Nrf2, PI3K/AKT/mTOR, Sanhuang decoction

## Abstract

Traditional Chinese medicine (TCM) has from ancient times been applied in China for the treatment of breast cancer with its own unique theoretical system. Sanhuang decoction composed of astragalus membranaceus, prepared rhubarb, and rhizoma curcumae longae has traditionally been used for antioxidant stress, inflammatory reaction, and angiogenesis. However, the role and mechanism of Sanhuang decoction in breast cancer remains unknown. The present study demonstrated the antitumor activity of Sanhuang decoction against breast cancer xenografts in nude mice. Notably, Sanhuang decoction promoted severe necrosis and induced cell death. In addition, Sanhuang decoction obviously regulated the inflammation and oxidative stress. Despite these, Sanhuang decoction could increase the expression of Nrf2. Moreover, si‐Nrf2 exhibited the opposite effects compared with the Sanhuang decoction treatment group and reversed the antibreast cancer role of Sanhuang decoction. Further, Sanhuang decoction remarkably suppressed the expression of PI3K/AKT/mTOR signaling pathway. Taken together, Sanhuang decoction was firstly evaluated to possess potent antibreast cancer effect in vivo through regulation of inflammation and oxidative stress accomplished by up‐regulation of Nrf2 via PI3K/AKT/mTOR signaling pathway and Sanhuang decoction might be a powerful candidate formula for antibreast cancer.

## 1 INTRODUCTION

Breast cancer is one of the most common malignant tumors in women, and the incidence of breast cancer accounts for 31% in women's tumors. There are about 1.350 million women diagnosed as breast cancer every year in the world, and more than 330, 000 women die from breast cancer (DeSantis, Ma, Bryan, & Jemal, 2014; Ganz & Goodwin, 2015). In China, breast cancer has ranked first in the mortality rate of malignant tumors in women, with the highest incidence in large and medium cities, and the morbidity crowd displays the younger in average age trend gradually, which undoubtedly has a huge negative impact in the whole society and individual families (Denny et al., 2017). Thus, exploring effective drugs for breast cancer is a hot topic and urgent task in the medical field. Although surgery, chemotherapy, radiotherapy, and endocrine therapy have been widely used in clinical practice, chemotherapy and radiotherapy have great damage effect to normal cells while killing tumor cells, and endocrine therapy also has its limitations (Hickey, Francis, & Lehman, 2013; Matsen & Neumayer, 2013; Reinbolt et al., 2015). Therefore, finding a way to improve the quality of life of patients is particularly important in the treatment and prevention of breast cancer.

Traditional Chinese medicine has its own characteristics and advantages in the treatment for the recurrence and metastasis of breast cancer (Wang, Xu, & Shen, 2015). In recent years, the clinical scientific research of Chinese medicine for breast cancer has tended to be rational and more closely connected with modern science. There have been many reports on the prevention and treatment of breast cancer from the aspects of cell and gene, which widens the research field of traditional Chinese medicine in treating breast cancer (Lee et al., 2014; Wong, Tan, Chen, Teo, & Chan, 2014). Many related studies have shown that the mechanism of traditional Chinese medicine for breast cancer treatment is eliminating stagnation, detumescence, dissipation blood stasis, acesodyne, dilatation of blood vessels, and improvement of microcirculation (Lin et al., (2018); Tsai, Lai, Lo, Chen, & Lin, 2017; Wang et al., 2016). Although the related research in China has made some progress, it is relatively backward relative to the research level of modern medicine. With the in‐depth study of the cause of the breast cancer in modern medicine, the mechanism of the prevention and cure of recurrence and metastasis of breast cancer through gene technology and molecular biology will be the trend of the breast cancer research in the future.

Sanhuang decoction is a prescription for the treatment of breast cancer in the mammary disease department of Jiangsu Province Hospital of TCM and is composed of three traditional Chinese herbs, including astragalus membranaceus, prepared rhubarb, and rhizoma curcumae longae concise compatible with 3:1: 1 (decocted in water) (Meng et al., (2017)). The clinical study showed that Sanhuang decoction can obviously improve the clinical symptom score of oxidative stress in patients, reduce the level of serum IL‐6, malondialdehyde, and tumor necrosis factor, and increase the level of superoxide dismutase. In addition, Sanhuang decoction has the positive effect of antioxidant stress, inflammatory reaction, and angiogenesis (Wang, Zhou, Zhai, Wang, & Chen, 2014). However, the role and possible mechanism of Sanhuang decoction in breast cancer have not been uncovered yet.

Nrf2, a transcription factor found in 1994, belongs to the CNC transcription factor family and is a soluble protein in the cytoplasm (Moi, Chan, Asunis, Cao, & Kan, 1994). In addition, Nrf2 is a receptor for the exogenous toxic substances and oxidative stress, which plays an important role in the main defense mechanisms involved in cellular antioxidant stress and exogenous toxic substances. Moreover, emerging evidences have reported that Nrf2 is aberrantly expressed in many tumors including breast cancer, lung cancer, gastric cancer, and so on (Hartikainen et al., 2012; Hayden et al., 2014; Hu, Yao, & Gao, 2013).

Therefore, the present study was designed to explore the role and possible function mechanism of Sanhuang decoction in the development and progression of breast cancer.

## 2 MATERIALS AND METHODS

### 2.1 Experimental materials

Herbs in Sanhuang decoction were purchased from Beijing Tongrentang drugstore and verified according to the Chinese pharmacopeia (2015). Nrf2 small interfering RNAs (siRNAs) were designed and synthesized by GenePharma Company.

### 2.2 Cell lines and cell culture

The human breast cancer cell MCF‐7 was obtained from American Type Tissue Culture Collection (Manassas, VA) and cultured in RPMI‐1640 medium (Invitrogen) supplemented with 10% fetal bovine serum (FBS, Invitrogen), 100 U/ml penicillin, and 100 μg/ml streptomycin (Sigma‐Aldrich) in a humidified cell incubator with 5% CO_2_ at 37ºC.

### 2.3 Xenograft tumors in nude mice

Female nude mice (6 weeks old, 18–22 g) were provided by Nanjing Medical University and were carried out according to the ethical guidelines by Nanjing Medical University. MCF‐7 cells (5 × 10^5^ cells in 30 µl) suspended in PBS were subcutaneously injected into the right axilla of the nude mice. Then, the mice were treated with Sanhuang decoction, si‐Nrf2, or Sanhuang decoction combined with si‐Nrf2 every 2 days for a total of 4 week. The mice of NC group were treated with PBS. Then, the mice were sacrificed. The weight of mice and tumors were calculated, and the tumor volume was calculated according to the formula, tumor volume = length × width^2^ × 0.5.

### 2.4 Enzyme‐linked immunosorbent assay (ELISA)

The ELISA kits (eBioscience) were performed to detect the levels of the cytokines, including TNF‐α and IL‐6 in serum according to the protocols provided by the manufacture. Cytokine levels were tested by measuring absorbance at 450 nm with a microplate reader.

### 2.5 Measurement of antioxidant enzyme activities

The serum was collected by centrifugation. The enzymatic activities, including superoxide dismutase (SOD), malondialdehyde (MDA), and total antioxidant capacity (T‐AOC), were measured in serum by using available kits according to the manufacturer's instructions.

### 2.6 Detection of ROS level

The level of ROS in serum was measured using a ROS assay kit (Beyotime) according to the manufacturer's protocol and analyzed by flow cytometry.

### 2.7 Quantitative real‐time PCR (qRT‐PCR)

Total RNA from tissues was extracted with TRIzol reagent (Invitrogen) according to the manufacturer's protocol, and complementary DNA was synthesized by a Prime Script RT kit (Takara Biotechnology Co., Ltd.). The relative expression was quantified using an ABI 7,500 Real‐Time PCR system (Applied Biosystems; Thermo Fisher Scientific, Inc.) with the SYBR Green PCR kit (Takara Biotechnology Co., Ltd). The levels were normalized to those of U6 using the 2^−ΔΔCt^ method.

### 2.8 Hematoxylin–eosin staining

The tumor tissues were excised and washed with in phosphate buffer saline (PBS), fixed in 4% formaldehyde, embedded in paraffin and sectioned (5 µm), and eventually stained with hematoxylin–eosin (H&E) for visual analysis.

### 2.9 Terminal deoxynucleotidyl transferase‐medimed dUTP nick‐end‐labeling (TUNEL) assay

TUNEL assay was performed to evaluate the apoptosis of tumor sections according to the manufacturer's protocol (Roche). Tumor sections were dewaxed and permeabilized with proteinase K for 15 min at room temperature. Sections were treated with 3% H_2_O_2_ to block endogenous peroxidases and incubated with equilibration buffer and terminal deoxynucleotidyl transferase enzyme. Finally, sections were incubated with antidigoxigenin–peroxidase conjugate. Tissue peroxidase activity was evaluated through DAB application. Sections were examined under a light microscope.

### 2.10 Immunohistochemistry assay

The tumor samples stained for Nrf2, PI3K, AKT, mTOR, VEGF, MMP‐2, and MMP‐9 were analyzed by immunohistochemical assays. Formalin‐fixed paraffin‐embedded tissue specimens were divided into 3–4 μm. After dewaxing and rehydrating, sections were submerged in hydrogen peroxide to quench peroxidase activity following incubated with 1% BSA to block nonspecific binding sites. After incubation with primary antibody at 4°C for 12 hr, secondary antibody was applied to slides for 1 hr at 25°C, and then, the sections were visualized using diaminobenzidine (DAB, Beyotime) under a light microscope (FSX100; Olympus).

### 2.11 Statistical analysis

GraphPad Prism 6 software was performed to carry out all statistical analysis. When only two groups were compared, Student's *t* test was conducted. One‐way analysis of variance was applied to compare difference between multiple groups. A value of *p* < .05 indicated that the difference was statistically significant.

## 3 RESULTS

### 3.1 Sanhuang decoction inhibited the development of MCF‐7 cancer xenografts in vivo

**Figure 1:**
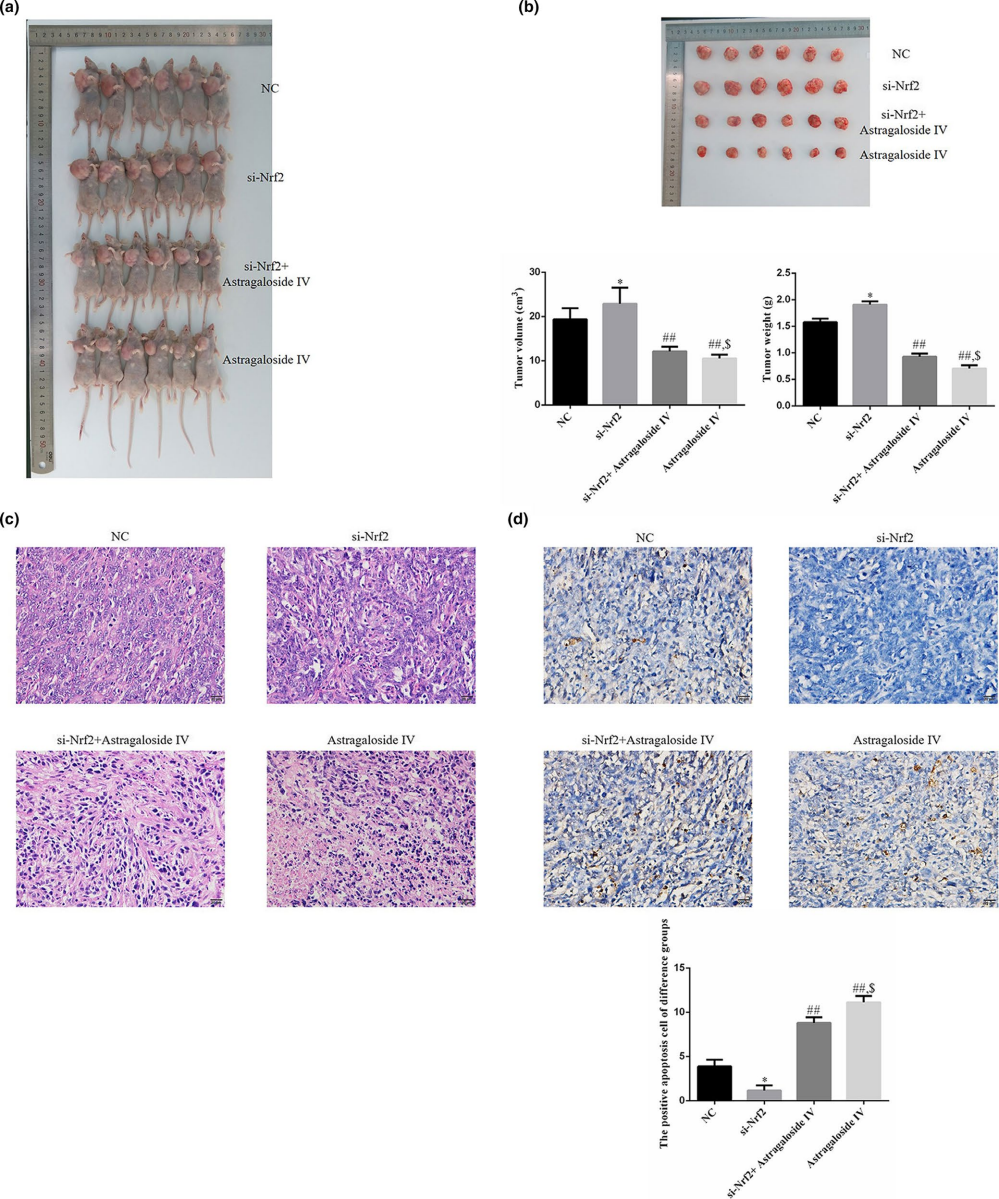
The tumor volume, weight, pathology, and cell apoptosis in vivo. NC: planting by normal MCF‐7 cell; si‐Nrf2: planting by MCF‐7 which was transfected with si‐Nrf2; si‐Nrf2 + Sanhuang: planting by MCF‐7 which was transfected with si‐Nrf2 and fed by Sanhuang decoction (4 g/kg); Sanhuang: planting by normal MCF‐7 cell and fed by Sanhuang decoction (4 g/kg). (a) The tumor of difference groups in body. (b) The tumor tissues taken out and tumor volume and weight. *: *p* ＜.05 versus NC; ##: *p *＜ .01 versus si‐Nrf2; $: *p* ＜ .05 versus si‐Nrf2 + Sanhuang. (c) The tumor pathology of difference groups by H＆E staining (400×). (d) The positive apoptosis cell of difference groups by TUNEL assay (400×). *: *p *＜ .05 versus NC; ##: *p* ＜ .01 versus si‐Nrf2; $: *p *＜ .05 versus si‐Nrf2 + Sanhuang

MCF‐7 cell transplanted xenografts tumor model of nude mice was performed to evaluate the antitumor role of Sanhuang decoction. The mice were treated with Sanhuang decoction (6.4 g/kg), si‐Nrf2, and combination of Sanhuang decoction with si‐Nrf2 every two days for a total period of four weeks. Then, the tumors from different groups were removed and weighted. As shown in Figure 1a, Sanhuang decoction could significantly reduce the tumor weight, while si‐Nrf2 could increase the tumor weight compared with NC group. In addition, obvious tumor suppress of tumor growth was observed in the groups treated with Sanhuang decoction, and si‐Nrf2 remarkably promoted the tumor growth (Figure 1b). Moreover, H & E staining assay was performed to examine the necrosis and infiltration of tumor tissues. As shown in Figure 1c, Sanhuang decoction promoted more severe necrosis and inhibited the infiltration in tumor tissues relative to those of NC group. Besides, the results of Figure 1d demonstrated the Sanhuang decoction treatment could notably induce cell death in vivo. However, si‐Nrf2 revealed the opposite effects when compared with the Sanhuang decoction treatment group. Further, si‐Nrf2 could reverse the antitumor effects of Sanhuang decoction. Taken together, Sanhuang decoction inhibited the tumor growth maybe through promotion of Nrf2.

### 3.2 Sanhuang decoction regulated expressions of inflammation factors

Numerous studies have shown that inflammatory factors can promote tumor growth and metastasis by changing the biological behavior of tumor cells and activating the stromal cells in the tumor microenvironment, such as vascular endothelial cells, tumor‐related macrophages, and fibroblasts (Ben‐Baruch, 2006; Coussens & Werb, 2002; Diakos, Charles, McMillan, & Clarke, 2014).

**Figure 2:**
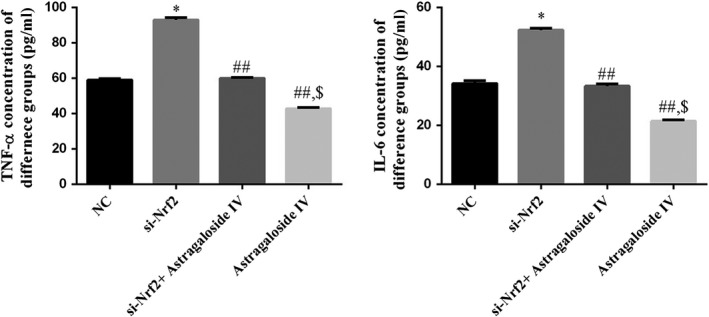
The TNF‐α and IL‐6 concentrations of difference groups by ELISA. *: *p* ＜ .05 versus NC; ##: *p* ＜ .01 versus si‐Nrf2; $: *p* ＜ .05 versus si‐Nrf2 + Sanhuang

To determine whether Sanhuang decoction effectively regulated the expressions of inflammation factors including TNF‐α and IL‐6, ELISA was carried out to examine the expressions of inflammation factors in serum after treatment. The result showed Sanhuang decoction could obviously inhibit the secretion of TNF‐α and IL‐6, while si‐Nrf2 promoted the levels of TNF‐α and IL‐6, and rescue the effects of Sanhuang decoction on the expressions of TNF‐α and IL‐6 (Figure 2).

### 3.3 Sanhuang decoction regulated the activities of antioxidant enzymes and ROS production

**Figure 3:**
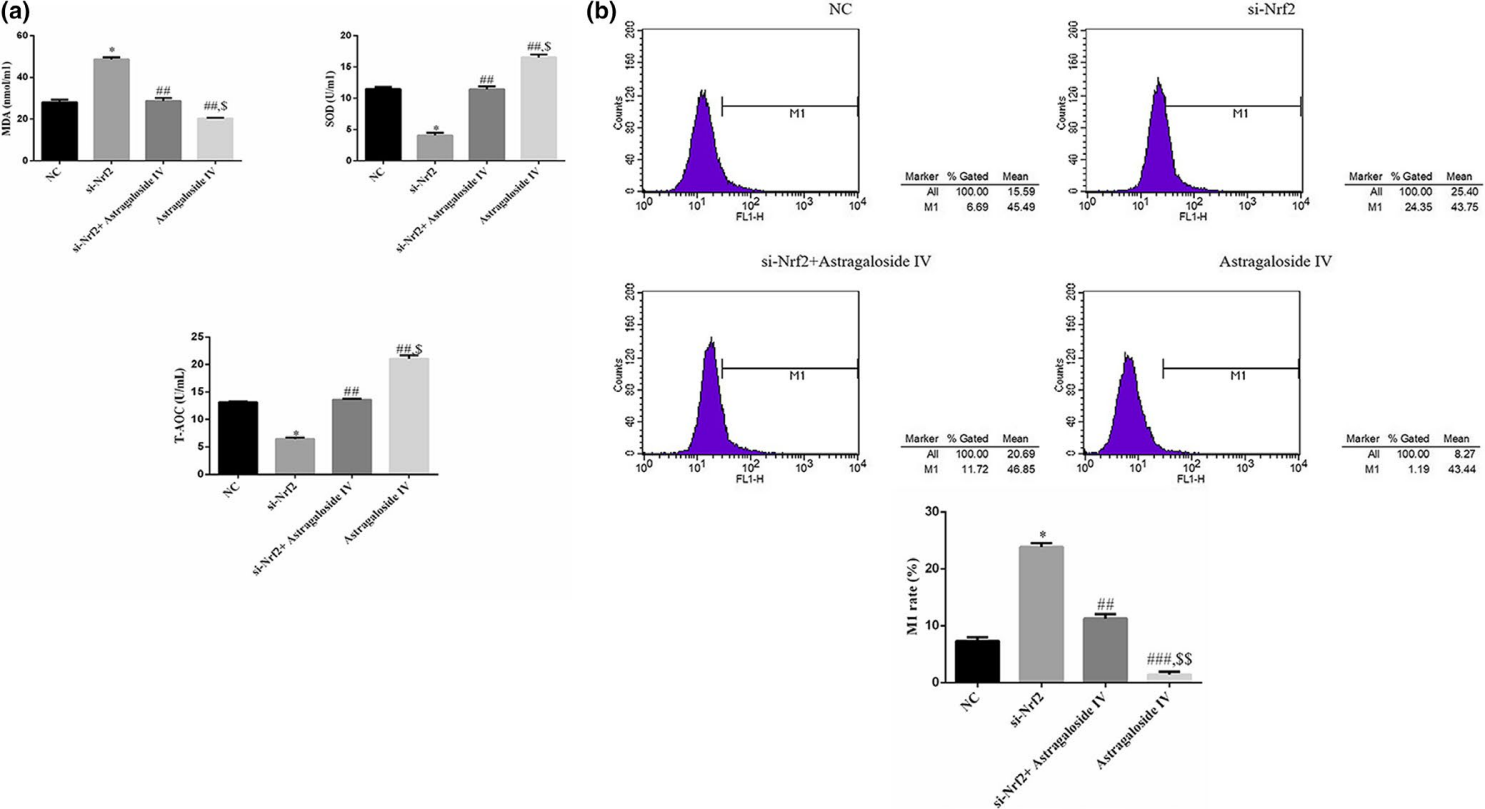
Antioxidant enzymes activities and ROS production. (a) The antioxidant enzyme activities of difference groups. *: *p* ＜ .05 versus NC; ##: *p* ＜ .01 versus si‐Nrf2; $: *p* ＜ .05 versus si‐Nrf2 + Sanhuang. (b) The ROS production of difference groups. *: *p* ＜ .05 versus NC; ##:*p *＜ .01 versus si‐Nrf2; $: *p* ＜ .05 versus si‐Nrf2 + Sanhuang

Further, the activities of antioxidant enzymes in serum, including superoxide dismutase (SOD), total antioxide capacity (T‐AOC), and malondialdehyde (MDA), were evaluated. The result indicated that Sanhuang decoction notably decreased the activities of SOD and T‐AOC and suppressed MDA compared to the NC group. However, treatment with si‐Nrf2 showed remarkably decreasing activities and could reverse the positive role of Sanhuang decoction as shown in Figure 3a.

To analyze the production of reactive oxygen species (ROS) in serum, flow cytometry was performed to detect the ROS content in serum. The results of Figure 3b showed that Sanhuang decoction obviously suppressed the production of ROS, while si‐Nrf2 promoted the production of ROS and could rescue the ROS inhibition of Sanhuang decoction.

### 3.4 Sanhuang decoction suppressed the mRNA and protein levels of VEGF, MMP‐2, and MMP‐9

**Figure 4:**
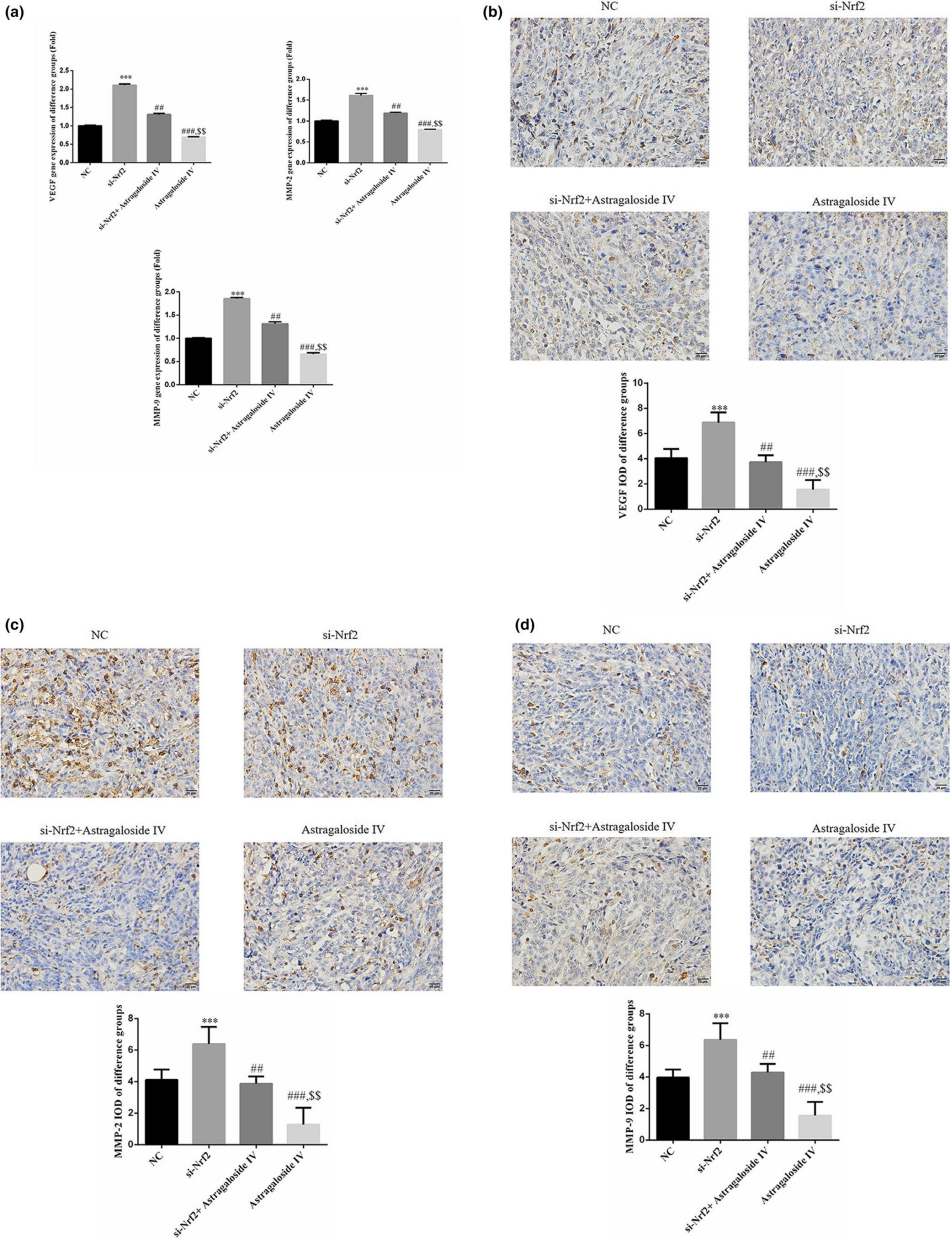
The mRNA and protein expressions of VEGF, MMP‐2, and MMP‐9. (a). The VEGF, MMP‐2, and MMP‐9 genes expressions of difference groups by RT‐PCR. ***: *p* ＜ .001 versus NC; ##: *p* ＜ .01 versus si‐Nrf2; $$: *p *＜ .01 versus si‐Nrf2 + Sanhuang. (b) The VEGF protein expression of difference groups by IHC (400×). ***: *p *＜ .001 versus NC; ##: *p *＜ .01 versus si‐Nrf2; $$: *p *＜ .01 versus si‐Nrf2 + Sanhuang. (c) The MMP‐2 protein expression of difference groups by IHC (400×). ***: *p *＜ .001 versus NC; ##:* p *＜ .01 versus si‐Nrf2; $$: *p *＜ .01 versus si‐Nrf2 + Sanhuang. (d) The MMP‐9 protein expression of difference groups by IHC (400×). ***: *p *＜ .001 versus NC; ##: *p *＜ .01 versus si‐Nrf2; $$: *p *＜ .01 versus si‐Nrf2 + Sanhuang

The aberrant expression of VEGF, MMP‐2, and MMP‐9 was closely related to the growth and metastasis of cancer. Thus, qRT‐PCR and immunohistochemistry assays were conducted to examine the mRNA and protein levels of VEGF, MMP‐2, and MMP‐9. As shown in Figure 4a,b, the expression of VEGF, MMP‐2, and MMP‐9 was observed to be down‐regulated in Sanhuang decoction treatment group, and si‐Nrf2 could obviously increase the expression of VEGF, MMP‐2, and MMP‐9 and remarkably restore the effects of Sanhuang decoction.

### 3.5 Sanhuang decoction regulated the PI3K/AKT/mTOR signaling pathway

**Figure 5:**
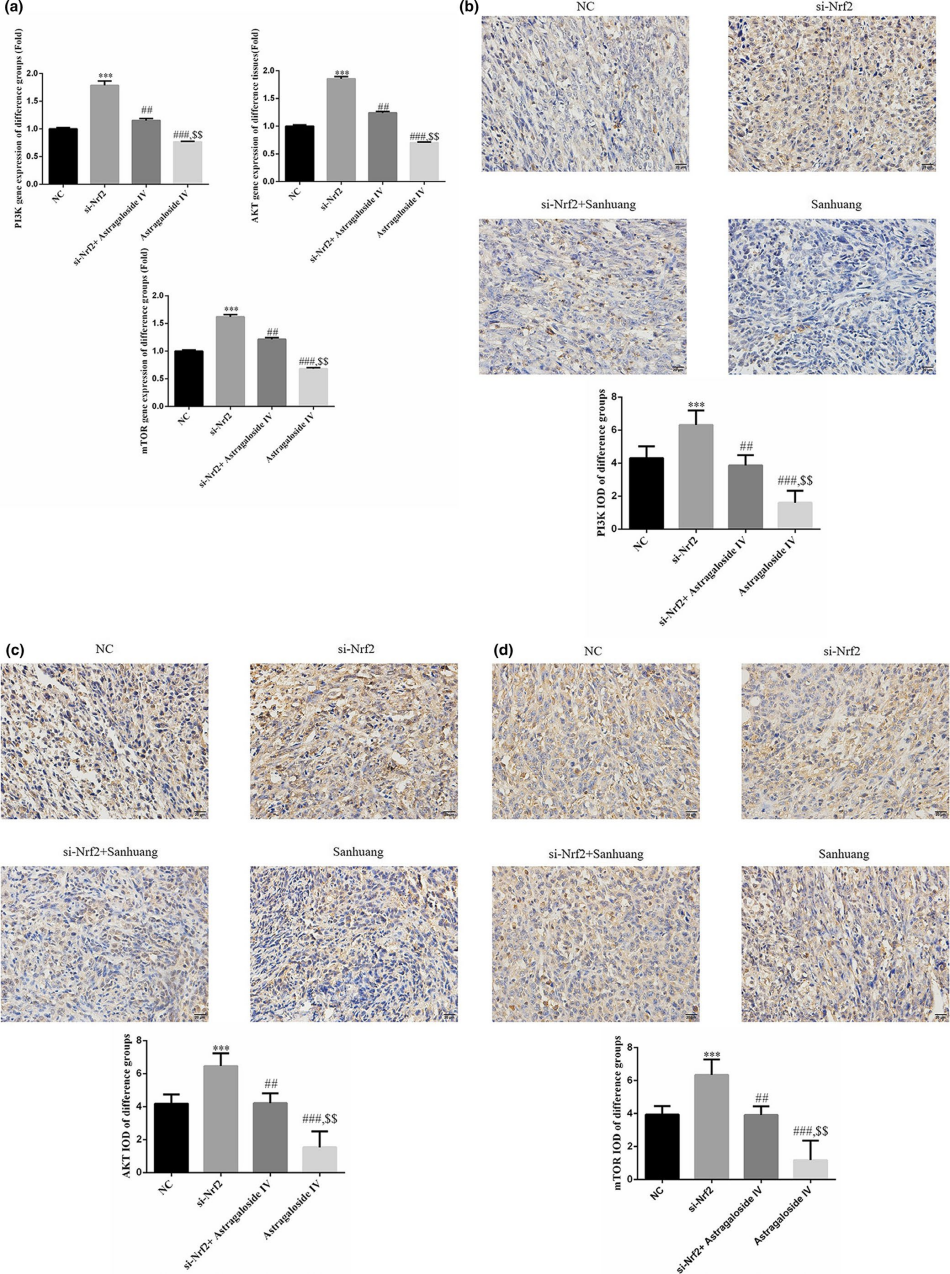
The mRNA and protein expressions of PI3K, AKT, and mTOR. (a) The PI3K, AKT, and mTOR gene expressions of difference groups by RT‐PCR. ***: *p *＜ .001 versus NC; ##:* p *＜ .01 versus si‐Nrf2; $$: *p *＜ .01 versus si‐Nrf2 + Sanhuang. (b) The PI3K protein expression of difference groups by IHC (400×). ***: *p *＜ .001 versus NC; ##: *p *＜ .01 versus si‐Nrf2; $$: *p *＜ .01 versus si‐Nrf2 + Sanhuang. (c) The AKT protein expression of difference groups by IHC (400×). ***: *p *＜ .001 versus NC; ##: *p *＜ .01 versus si‐Nrf2; $$: *p *＜ .01 versus si‐Nrf2 + Sanhuang. (d) The mTOR protein expression of difference groups by IHC (400×). ***: *p *＜ .001 versus NC; ##: *p *＜ .01 versus si‐Nrf2; $$: *p* ＜ .01 versus si‐Nrf2 + Sanhuang

The results above demonstrated that Sanhuang decoction had positive inhibitory effects on the MCF‐7 cancer xenografts maybe through promotion of Nrf2. Therefore, to further explore the possible mechanisms of Sanhuang decoction on breast cancer. The PI3K/AKT/mTOR pathway is a very attractive therapeutic target in clinical trials of cancer treatment, and activation of PI3K/AKT/mTOR is closely associated with cell proliferation, survival, and metastasis. Besides, the activation of PI3K/AKT/mTOR pathway is related to the expression of Nrf2 (Bartholomeusz and Gonzalez‐Angulo, (2012); Brazil, Park, & Hemmings, 2002; Tsai, Shen, et al., 2017). From the results of Figure 5a,b, the expression of PI3K, AKT, and mTOR was discovered to be significantly decreased after treatment with Sanhuang decoction, and si‐Nrf2 could notably promote the mRNA and protein levels of PI3K, AKT, and mTOR. In addition, cotreatment of Sanhuang decoction with si‐Nrf2, si‐Nrf2 could remarkably reverse the inhibitory effects of Sanhuang decoction on PI3K/AKT/mTOR pathway. These data suggested that Sanhuang decoction inhibited the PI3K/AKT/mTOR pathway maybe through regulation of Nrf2.

## 4 DISCUSSION

Breast cancer is one of the most common malignant tumors in women. In the western countries represented by the United States, breast cancer accounts for the first place in the incidence of cancer in all women with a second mortality rate (Siegel, Miller, & Jemal, 2017). WHO statistics show that about 460, 000 patients worldwide die from the breast cancer each year. Although the overall incidence of breast cancer in China is lower than that in western countries, it also presents a gradually increasing trend. In 2000, the incidence of breast cancer among Chinese women was 19.9/10 million, ranking the first in Chinese female malignant tumors (Chen et al., 2016; Yang, Parkin, Ferlay, Li, & Chen, 2005). Therefore, exploring effective drugs for breast cancer is a hot topic and urgent task in the medical field. Current therapy strategies for breast cancer focus on chemotherapy, radiotherapy, and so on, but these therapy strategies almost have disadvantages with single target, unsatisfactory curative effect, and side effects, which has become the bottleneck of clinical tumor therapy (Hickey et al., 2013; Matsen & Neumayer, 2013; Reinbolt et al., 2015). Nevertheless, the studies on medical research show that traditional Chinese herbal medicine is expected to become a tool to break through these bottlenecks. Based on the theory of traditional Chinese medicine and combined application of multiple components of Compound Chinese Traditional Medicine (CCTM) (Liu et al., 2017; Ni et al., 2014), the combination of nearly 100, 000 compounds has been used for more than 2, 500 years in clinical prevention and treatment of various diseases, and combined treatment of breast cancer with Chinese herbal compound has been proved effective clinically (Lee et al., 2014; Wong et al., 2014). Sanhuang decoction composed of astragalus membranaceus, prepared rhubarb, and rhizoma curcumae longae has been widely used for the treatment of antioxidant stress, inflammatory reaction, and angiogenesis in patients (Wang et al., (2014)). However, the role and possible mechanism of Sanhuang decoction in breast cancer have not been uncovered yet. Therefore, this study was designed to explore the antitumor effects of decoction. As expected, Sanhuang decoction had positive role in inhibiting the growth of MCF‐7 cancer xenografts in vivo, and the results of H & E and TUNEL demonstrated that Sanhuang decoction could promote severe necrosis and induce cell death. Besides, qRT‐PCR and immunohistochemistry assays were performed to evaluate the expression of VEGF, MMP‐2, and MMP‐9 related to the invasion and metastasis of cancer, and the results showed that Sanhuang decoction could significantly inhibited the expression of VEGF, MMP‐2, and MMP‐9. These data suggested that Sanhuang decoction had the antitumor effects of breast cancer.

With the in‐depth study of molecular biology of breast cancer, more and more scholars believe that the occurrence and development of breast cancer are not only related to the factors such as heredity and environment, but also closely related to the inflammatory state of the body (Jiang & Shapiro, 2014). The role of IL‐6 and TNF‐α in the adhesion, migration, and apoptosis of breast cancer cells has attracted more and more attention (Zeng et al., 2016). In the study, we detected the expression of inflammation factors in mice treated with Sanhuang decoction, and the result showed that the expression of IL‐6 and TNF‐α was observed to be obviously decreased compared with the NC group. ROS plays an important role in the occurrence and development of breast cancer (Brody et al., 2007). Long‐term low level of ROS can promote the proliferation of breast cancer cells, increase survival and tumorigenicity, up‐regulate the level of VEGF, and induce angiogenesis (Abdullah, Mohammed, Rasedee, & Mirghani, 2015). At the same time, ROS can promote the metastasis of breast cancer cells and even activate some signaling pathways, such as P13K/AKT pathway (Penney & Roy, 2013; Ushio‐Fukai & Nakamura, 2008). Under normal circumstances, the body's oxidation and antioxidant system are dynamically balanced. Once oxidative and antioxidant mechanisms are destroyed, oxidative stress can be produced, leading to disease. MDA is a product of lipid peroxidation, and its content directly reflects the degree of oxidative stress. SOD is an important antioxidant enzyme in the body to eliminate superoxide anion, and its activity reflects the antioxidant capacity of enzymes in vivo. The levels of enzymes and nonenzymatic reductive substances constitute the total antioxidant capacity of the body (Stanojkovic et al., 2011). Therefore, the increase in MDA and the decrease in antioxidants (SOD and T‐AOC) reflect the degree of imbalance of oxidation and oxidation, which is closely associated with the development and progression of breast cancer (Gupta et al., 2012; Kilic, Yavuz Taslipinar, Guney, Tekin, & Onuk, 2014). The data revealed that Sanhuang decoction could significantly suppress the expression of ROS, MDA, and T‐AOC and notably promote the expression of SOD. These results indicated that the antitumor role of Sanhuang decoction may be closely related to the regulation of inflammation and oxidative stress.

At present, research on Nrf2 in chemical defense of cancer is still in the ascendant. Study Nrf2 deletion or activation disorder can induce cell sensitivity to stimulation, which is closely related to the prolongation of the process of inflammation repair, cell apoptosis, and so on (Kobayashi, Ohta, & Yamamoto, 2004). Recent studies have shown that Nrf2 can enhance the tolerance of tumor cells to chemotherapeutic drugs and promote the growth of tumor cells. In nonsmall lung cancer cells, silencing Nrf2 gene expression by RNAi can inhibit the growth of tumor cells (Singh et al., 2008). In addition, the expression of Nrt2 can inhibit the formation of lung cancer and prostate cancer induced by oxidative stress (Barve et al., 2009; Harvey et al., 2009). In breast cancer cells, high expression of Nrf2 can enhance the ability of cells to resist oxidative damage induced by H_2_O_2_ (Hsieh, Elangovan, & Wu, 2010). Moreover, previous studies have also illustrated some of the functions of Nrf2 in preventing oxidative stress induced breast cancer (Klaunig, Kamendulis, & Hocevar, 2010; Kwak & Kensler, 2010). To further evaluate whether Sanhuang decoction could affect the expression of Nrf2, we used si‐Nrf2 and combination of Sanhuang decoction with si‐Nrf2 to treat the mice, and the results revealed that Sanhuang decoction could increase the expression of Nrf‐2. Besides, Nrf2 play an important role in the growth and metastasis of breast cancer might through regulation of inflammation and oxidative stress. In addition, si‐Nrf2 could reverse the effect of Sanhuang decoction on breast cancer.

PI3K pathway is a signal transduction pathway that participates in the regulation of cell growth, proliferation, and differentiation and plays an important role in the occurrence, development, treatment, and prognosis of malignant tumors (Fruman & Rommel, 2014). Over the years, PI3K signaling pathway has also been an important pathway in breast cancer research (Fry, 2001; Rexer & Arteaga, 2013). AKT is a kind of Ser/Thr protein kinases and is the downstream effect gene of PI3K, which is closely related to the occurrence and development of malignant tumor (Ma & Hu, 2013). AKT is considered to be the key step in the PI3K‐AKT‐mTOR signal transduction pathway. Phosphorylation of AKT can activate AKT, and activated AKT regulates a series of physiological processes of cells through regulating the substrate proteins. Abnormal activation of AKT can occur in many malignant cells (Cassinelli et al., 2013). Mammalian target of rapamycin (mTOR) is an atypical Ser/Thr protein kinase and is a downstream effector of AKT. Activated AKT can participate in multiple signaling pathways and regulate transcription and protein synthesis via activating the downstream mTOR directly or through some cytokines to play an important role in the growth and proliferation of tumor cells. PI3K‐AKT‐mTOR signal transduction pathway controls a large number of tumor markers involving many functions such as cell cycle, cell survival, metabolism, cell movement, and genomic instability (Ekim et al., 2011; Showkat, Beigh, & Andrabi, 2014). In the present study, we found Sanhuang decoction could significantly inhibit the expression of PI3K/AKT/mTOR pathway, while si‐Nrf2 obviously promoted the expression of PI3K/AKT/mTOR pathway and reverse the effects of Sanhuang decoction.

Taken together, Sanhuang decoction was firstly evaluated to possess potent antibreast cancer effect in vivo through regulation of inflammation and oxidative stress accomplished by up‐regulation of Nrf2 via PI3K/AKT/mTOR signaling pathway. Therefore, Sanhuang decoction might be a powerful candidate formula for antibreast cancer.

## CONFLICT OF INTEREST

None declared.

## ETHICAL STATEMENT

This study was approved by Ethics Committee of the First Affiliated Hospital of Nanjing University of Traditional Chinese Medicine.
